# Situs Inversus Totalis on ^18^F-FDG PET/CT: A Case Report and a Literature Review

**DOI:** 10.3389/fmed.2022.840795

**Published:** 2022-03-09

**Authors:** Xu-Sheng Liu, Rui-Min Wu, Hua-Bing Wan, Yi-Jia Chen, Fan Tan, Dao-Bing Zeng, Yi Yang, Zhi-Jun Pei

**Affiliations:** Department of Nuclear Medicine and Institute of Anesthesiology and Pain, Taihe Hospital, University of Medicine, Shiyan, China

**Keywords:** 18 F-FDG, PET/CT, small cell lung cancer, situs inversus totalis, neuroendocrine carcinoma

## Abstract

A 62-year-old female patient with pathologically confirmed left lung small cell neuroendocrine carcinoma. The patient was referred to our positron emission tomography (PET)/CT center to look for possible metastatic diseases. After fasting for 8 h, the fasting blood glucose level of the patient was 7.1 mmol/L. The patient was intravenously injected with a 6.42 mCi (238 MBq) ^18^F-fluorodeoxyglucose (FDG) imaging agent. After the patient rested for 1 h, we scanned the patient with SIEMENS Biograph mCT 64 PET/CT camera. In addition to lung tumors and lymph node diseases, abnormal tracer uptake in the patient's thyroid was also found. PET/CT also showed situs inversus totalis of the patient, including the dextrocardia, liver on the left side, stomach, and spleen on the right side of the patient's body. The identification of anatomical variations and abnormalities by PET/CT imaging is very important to develop the best treatment for lung cancer.

## Introduction

Situs inversus totalis refers to the position of the thoracic cavity, abdominal cavity, or thoracic and abdominal cavity, which is opposite to normal and presents a mirror image with healthy people (1). This kind of patient is rare in clinics (2). It is reported that situs inversus totalis (SIT) can be associated with multiple cancers, such as breast cancer (3), esophageal cancer (4), and cervical cancer (5). Lung small cell neuroendocrine carcinoma, also known as small cell lung cancer (SCLC), is a subtype of pulmonary neuroendocrine tumors (PNETs) (6). According to the classification of who in 2015, PNETs are divided into four subtypes: typical carcinoid (TC), atypical carcinoid (AC), large cell neuroendocrine carcinoma (LCNEC), and SCLC (7). PNETs are epithelial tumors derived from pulmonary neuroendocrine cells. They have unique biological and clinical characteristics, accounting for about 20% of primary lung tumors. They are a common type of neuroendocrine tumor (NETs) (8). It is rare for the same patient to have both SIT and SCLC. Positron emission tomography (PET)/CT is a molecular imaging technology able to provide both anatomical and functional information. It can obtain cross-sectional images of the whole body in all directions, to achieve the purpose of early detection of lesions and diagnosis of diseases (9). In this study, we reported the PET/CT of patients with SIT along with SCLC as follows.

## Case Presentation

A 62-year-old woman was admitted with a cough with intermittent coughed blood. The main performance is no obvious inducement of cough, mainly obvious at night, the weight loss of 10 kg within half a month. The patient's cough has worsened in the past day with hemoptysis. CT scan showed central lung cancer with obstructive pneumonia in the lower lobe of the left lung, and SIT was also found. The serum ferritin level was 300.73 ng/ml and the carbohydrate antigen CA125 level was 132.8 μ/ml. The pathological results of bronchoscopy biopsy confirmed that it was small cell neuroendocrine carcinoma of the lung [immunohistochemical results: CK (P) (+), CD56 (+), Cg-A (+), CK7 (–), P63 (–), P40 (–), Ki-67 (90%+), TTF-1(SPT24) (+), and Syn (+)]. The patient was referred to our department for an ^18^F-fluorodeoxyglucose (FDG) PET/CT examination.

**Figure 1:**
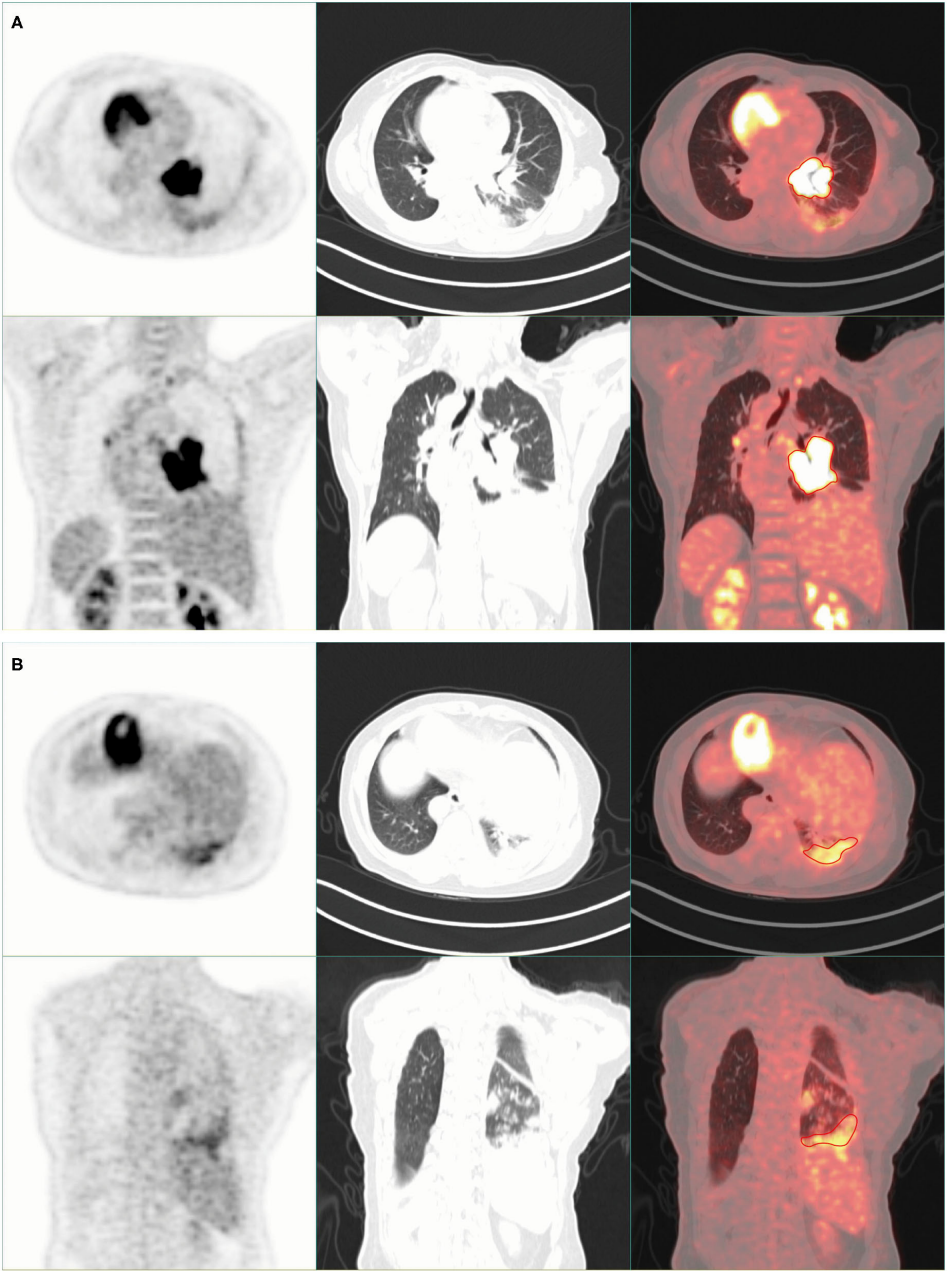
Chest imaging of positron emission tomography (PET)/CT scan. **(A)** There was high tracer uptake in the left hilar area. **(B)** The bronchus of the lower lobe of the left lung was truncated, and high tracer uptake was observed.

**Figure 2:**
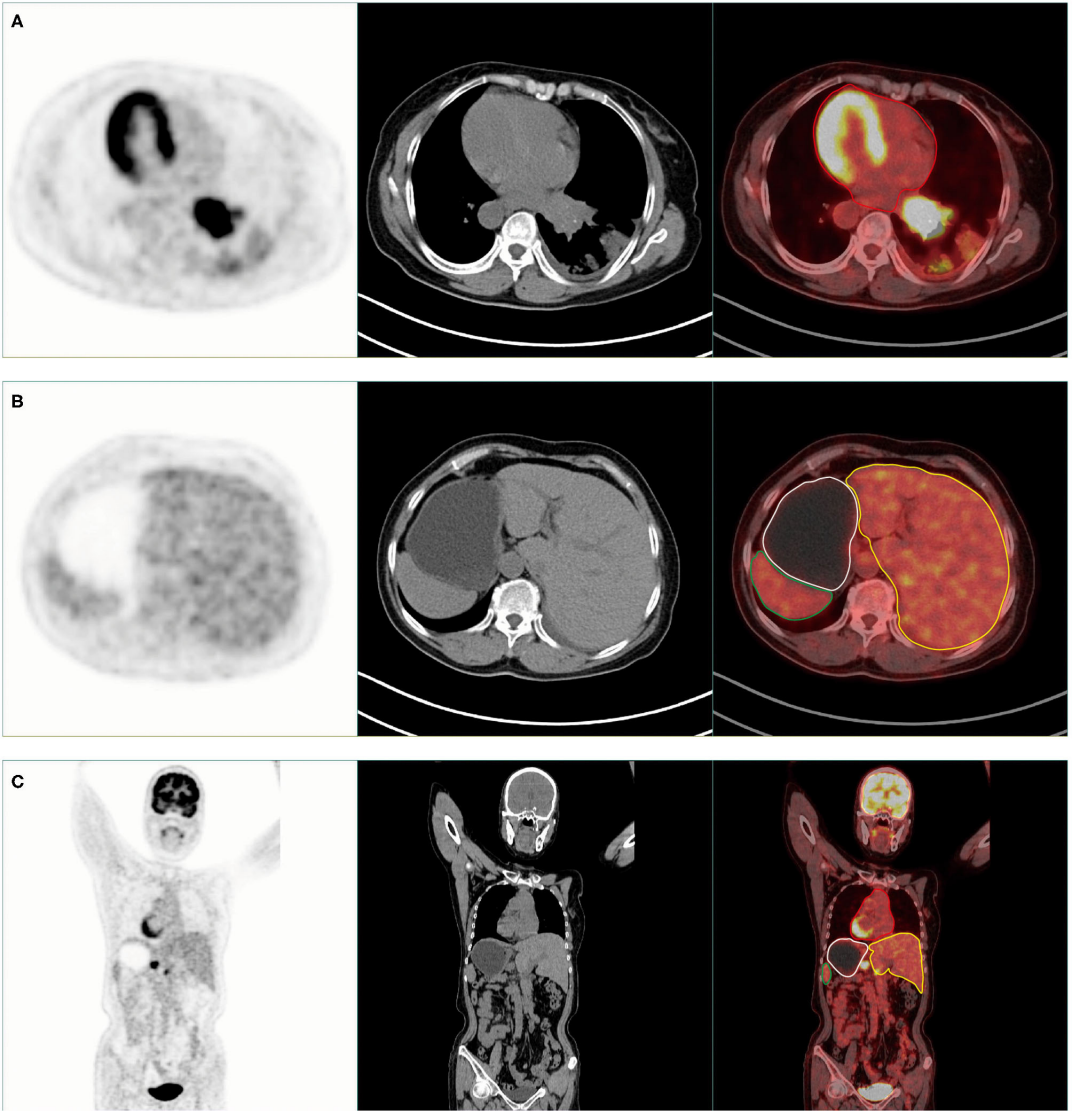
PET/CT scan showed that this was a patient with situs inversus totalis (SIT). **(A,B)** Axial PET, CT, and PET/CT fusion images show the dextrocardia (red area), liver on the left side (yellow area), stomach (white area), and spleen (green area) on the right side of the patient's body. **(C)** Coronal PET, CT, and PET/CT fusion images show the dextrocardia (red area), liver on the left side (yellow area), stomach (white area), and spleen (green area) on the right side of the patient's body.

The PET/CT (SIEMENS Biograph, Germany) scan results showed high tracer uptake in the left hilar area (Figure 1A). The bronchus of the lower lobe of the left lung was truncated and significant uptake of tracer was observed (Figure 1B). The results of this scan were consistent with those of CT scan and pathological examination. An interesting phenomenon was found when observing the whole-body PET/CT scanning images of patients. PET/CT scan showed that this was a patient with SIT. The imaging of the heart, liver, stomach, and other organs in the patient is completely opposite to that in the normal person (Figure 2).

**Figure 3:**
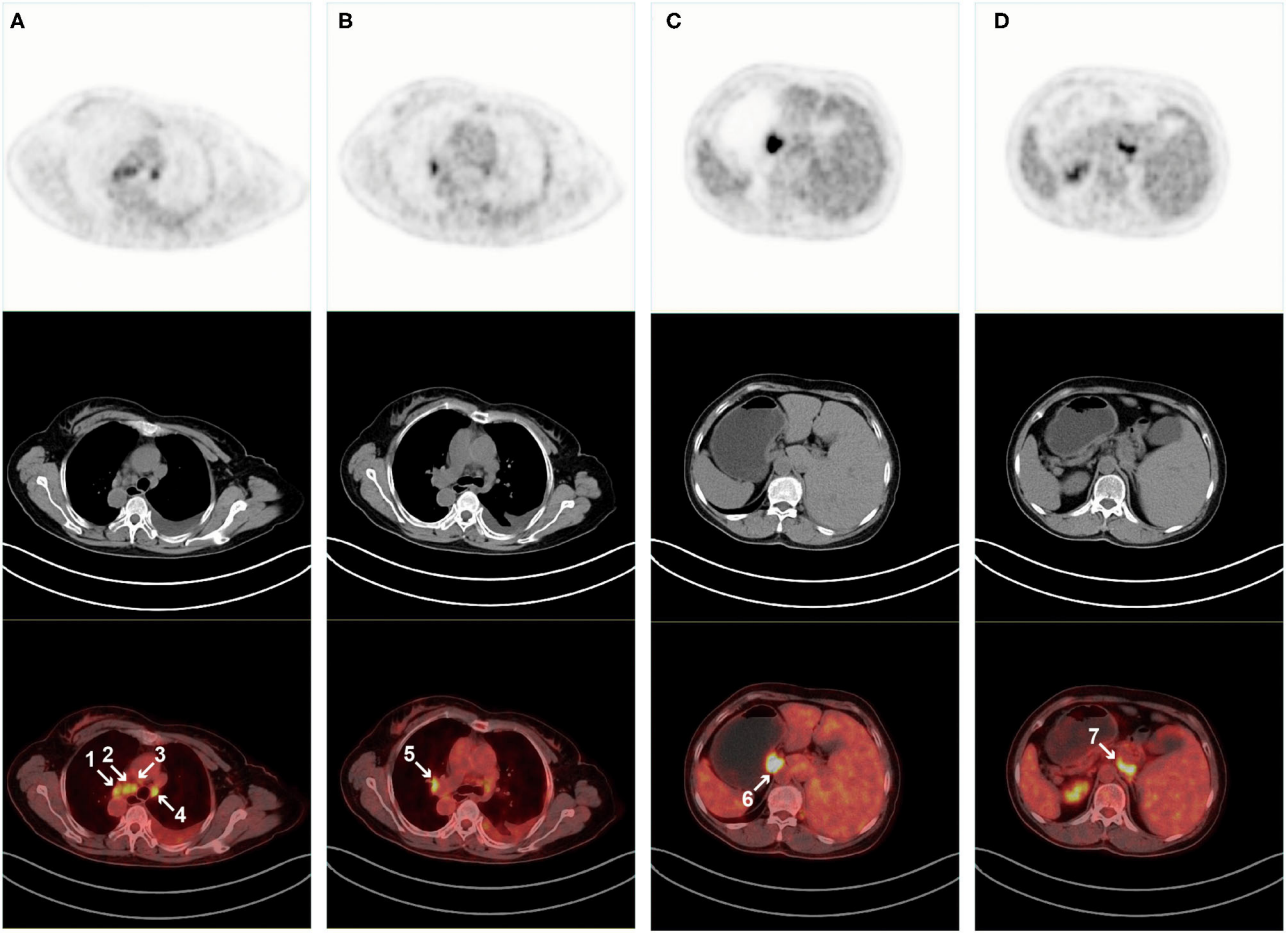
PET/CT scan found that multiple lymph nodes also had high tracer uptake. Axial and coronal PET, CT, and PET/CT fusion images show tracer uptake in the mediastinum **(A)** Right hilar, **(B)** Gastric, **(C)** Liver, and **(D)** Lymph nodes.

**Figure 4:**
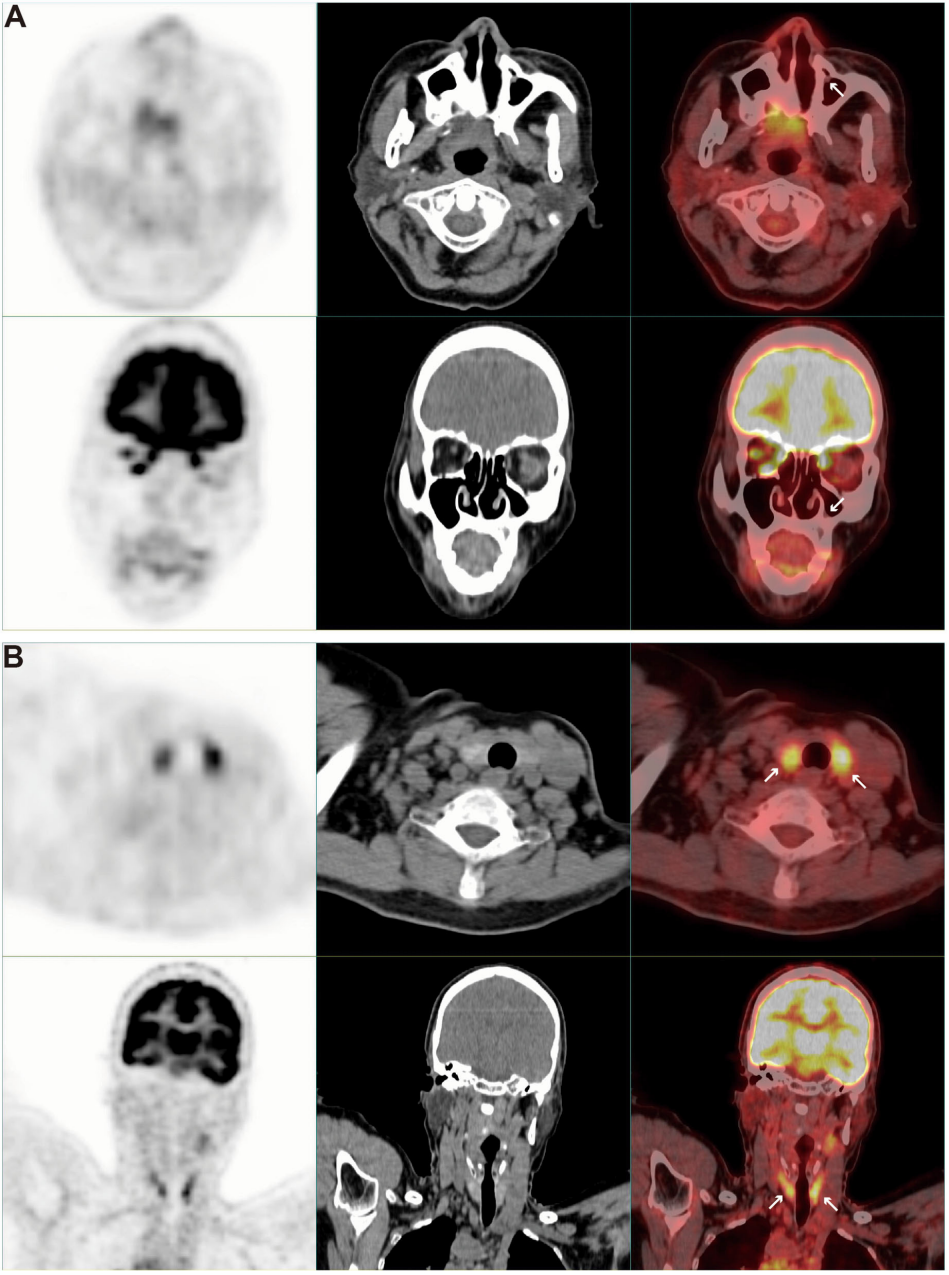
PET / CT scan showed abnormalities of maxillary sinus and thyroid. **(A)** Axial and coronal PET, CT, and PET/CT fusion images show mucinous changes in the left maxillary sinus. **(B)** Axial and coronal PET, CT, and PET/CT fusion images showed abnormal tracer uptake in the patient's thyroid.

In addition to the known high tracer uptake in left lung tumors, PET/CT scan also found high tracer uptake in multiple lymph nodes. These lymph nodes were distributed in the mediastinum (Figure 3A), right hilum (Figure 3B), stomach (Figure 3C), and liver (Figure 3D). At the same time, we also found mucinous changes in the patient's left maxillary sinus, and its tracer uptake was slightly increased (Figure 4A). However, PET/CT showed no significant change in thyroid density, but the tracer uptake was abnormally increased (Figure 4B). However, the levels of thyroid-stimulating hormone (TSH, 4.57 mIU/L, 0.4–5), triiodothyronine (T3, 1.83 nmol/L, 1.07–2.6), thyroxine (T4, 117.46 nmol/L, 69–161), free triiodothyronine (FT3, 4 pmol/L, 2.76–7.65), and free thyroxine (FT4, 8.09 pmol/L, 7.98–16.02) in the patient's serum were within the normal range. Laboratory examination also found that the contents of alanine aminotransaminase (ALT, 47.3 U/L, 0–40), aspartate aminotransaminase (AST, 53.7 U/L, 0–35), γ-glutamyltransferase (GGT, 112 U/L, 0–45) and alkaline phosphatase (ALP, 157.6 U/L, 50–135) in the patient's serum were significantly increased, but PET/CT scan did not find obvious abnormalities in the liver.

## Discussion

Situs inversus totalis (SIT), is a rare congenital anomaly, represents a complete left to right side transposition of the asymmetrical thoracic and abdominal organs, and incorporates dextrocardia (1, 3). As shown in this case, simultaneous abnormalities in the respiratory and lymphatic system often complicate SIT cases. Interestingly, the occurrence of lung small cell neuroendocrine carcinoma in patients with SIT is extremely rare. This is the first reported case of SCLC, lymph node metastasis, and SIT.

Although surgical resection is a common method for SCLC treatment, it is more difficult to stage and treat SCLC after SIT and lymph node metastasis. PET/CT not only has the characteristics of high spatial resolution and clear anatomical structure of CT but also has the advantages of PET functional imaging. At the same time, PET/CT scan can also provide information about whether there is metastasis in various organs of the whole body and provide strong evidence for the accurate staging of tumors (9).

It is well known that SIT, bronchiectasis, and sinusitis are typical features of Kartagener syndrome. Kartagener syndrome complicated with lung cancer has also been reported before (10). Although there is no pathological evidence of bronchiectasis, in this case, careful examination is still needed before the operation to rule out the possibility of Kartagener syndrome. At the same time, if surgical treatment is required, surgeons need to be very careful about SIT to avoid errors. Although SIT has been reported with solid tumors (1, 3–5, 11), whether an association between SIT and malignancies has been controversial, and further clinical and epidemiological research evidence is needed due to objective limitations such as the number of cases. It is also necessary to see if malignant metastases already exist when developing treatment options for patients with SIT with tumors. PET/CT scanning is very important to identify the abnormal biological distribution of tracers and observe anatomical variation, so as to help clinicians formulate the best treatment strategy for patients.

## Data Availability Statement

The original contributions presented in the study are included in the article/supplementary material, further inquiries can be directed to the corresponding author/s.

## Ethics Statement

Written informed consent was obtained from the individual(s) for the publication of any potentially identifiable images or data included in this article.

## Author Contributions

X-SL conceived the project and wrote the manuscript. X-SL, R-MW, and H-BW performed image acquisition and prepared images. X-SL, Y-JC, FT, D-BZ, and YY participated in the discussion and language editing. Z-JP reviewed the manuscript. All authors contributed to the article and approved the submitted version.

## Conflict of Interest

The authors declare that the research was conducted in the absence of any commercial or financial relationships that could be construed as a potential conflict of interest.

## Publisher's Note

All claims expressed in this article are solely those of the authors and do not necessarily represent those of their affiliated organizations, or those of the publisher, the editors and the reviewers. Any product that may be evaluated in this article, or claim that may be made by its manufacturer, is not guaranteed or endorsed by the publisher.

## References

[1] Calabria FF Leporace M Bagnato A. Situs inversus totalis and cholangiocarcinoma of the gallbladder detected by 18F-FDG PET/CT. Clin Nucl Med. (2018) 43:439–40. 10.1097/RLU.0000000000002047 29538026

[2] Iwamura T Shibata N Haraguchi Y Hisashi Y Nishikawa T Yamada H. Synchronous Double Cancer of the Stomach and Rectum With Situs Inversus Totalis and Polysplenia. Syndrome. (2001) 12:148–53. 10.1097/00004836-200108000-00012 11468444

[3] Halac M Mut SS Ylmaz S Ergul N Sonmezoglu K. Appearance of Situs Inversus Totalis and Polysplenia Syndrome on FDG PET/CT. Clin Nucl Med. (2008) 33:142–3. 10.1097/RLU.0b013e31815ef836 18209543

[4] Xie CL Cai JS Tan ZH Yang J Yang HX. Total minimally invasive mckeown esophagectomy in an esophageal cancer patient with situs inversus totalis: a case report. Thorac Cancer. (2021) 12:122–7. 10.1111/1759-7714.13723 33155374 PMC7779195

[5] Zhang Y Chen Y Huang Z Zhou F. Adenocarcinoma of the cervix uteri and endometrium combined with the kartagener syndrome on FDG PET/CT. Clin Nucl Med. (2015) 40:922–3. PubMed PMID: 26284775. 10.1097/RLU.0000000000000961 26284775

[6] Rekhtman N. Lung neuroendocrine neoplasms: recent progress and persistent challenges. Modern Pathol. (2021) 21:943. 10.1038/s41379-021-00943-2 34663914 PMC8695375

[7] Travis WD Brambilla E Nicholson AG Yatabe Y Austin JHM Beasley MB. The 2015 world health organization classification of lung tumors: impact of genetic, clinical and radiologic advances since the 2004 classification. J Thorac Oncol. (2015) 10:1243–60. 10.1097/JTO.0000000000000630 26291008

[8] Sackstein PE O'Neil DS Neugut AI Chabot J Fojo T. Epidemiologic Trends in Neuroendocrine tumors: an examination of incidence rates and survival of specific patient subgroups over the past 20 years. Semin Oncol. (2018) 45:249–58. 10.1053/j.seminoncol.2018.07.001 30348533

[9] Rowe SP Pomper MG. Molecular imaging in oncology: current impact and future directions. CA: Cancer J Clinic. (2021) 21:21713. 10.3322/caac.21713 34902160 PMC9189244

[10] Zhou D Tian Y Lu Y Yang X. Anatomical variants of pulmonary segments and uni-portal thoracoscopic segmentectomy for lung cancer in a patient with kartagener syndrome: a case report. General Thoracic Cardiovascul Surg. (2021) 69:1432–7. 10.1007/s11748-021-01685-3 34283387 PMC8416861

[11] Huang J Yang H Wang M Zhao X Shao S Zhang F. Gallbladder adenosquamous cancer with situs inversus totalis: a case report and literature review. Oncotargets Ther. (2021) 14:4299–304. 10.2147/OTT.S319030 34349522 PMC8327361

